# Using ultrasound-targeted microbubble destruction to enhance radiotherapy of glioblastoma

**DOI:** 10.1007/s00432-021-03542-5

**Published:** 2021-02-06

**Authors:** Chanjuan Peng, Yong Wu, Yang Yang, Ningning Li, Xi Chen, Linhui Gu, Dong Xu, Chen Yang

**Affiliations:** 1 Department of Ultrasound in Medicine, Cancer Hospital of the University of Chinese Academy of Sciences (Zhejiang Cancer Hospital), Hangzhou, 310022 Zhejiang China; 2 Department of Medical Engineering, Cancer Hospital of the University of Chinese Academy of Sciences (Zhejiang Cancer Hospital), Hangzhou, 310022 Zhejiang China; 3 Department of Radiation Oncology, Cancer Hospital of the University of Chinese Academy of Sciences (Zhejiang Cancer Hospital), Hangzhou, 310022 Zhejiang China; 4 Department of Pathology, Cancer Hospital of the University of Chinese Academy of Sciences (Zhejiang Cancer Hospital), Hangzhou, 310022 Zhejiang China; 5 Department of Core Facility Service, Cancer Hospital of the University of Chinese Academy of Sciences (Zhejiang Cancer Hospital), Hangzhou, 310022 Zhejiang China; 6 Institute of Cancer and Basic Medicine (IBMC), Chinese Academy of Sciences, Hangzhou, 310022 Zhejiang China

**Keywords:** Ultrasound, Microbubbles, Ultrasound therapy, Radiation therapy, Glioblastoma

## Abstract

**Objective:**

To investigate the efficacy and mechanism of ultrasound-targeted microbubble destruction (UTMD) combined with radiotherapy (XRT) on glioblastoma.

**Methods:**

The enhanced radiosensitization by UTMD was assessed through colony formation and cell apoptosis in Human glioblastoma cells (U87MG). Subcutaneous transplantation tumors in 24 nude mice implanted with U87MG cells were randomly assigned to 4 different treatment groups (Control, UTMD, XRT, and UTMD + XRT) based on tumor sizes (100–300 mm^3^). Tumor growth was observed for 10 days after treatment, and then histopathology stains (HE, CD34, and γH2AX) were applied to the tumor samples. A TUNEL staining experiment was applied to detect the apoptosis rate of mice tumor samples. Meanwhile, tissue proteins were extracted from animal specimens, and the expressions of dsDNA break repair-related proteins from animal specimens were examined by the western blot.

**Results:**

When the radiotherapy dose was 4 Gy, the colony formation rate of U87MG cells in the UTMD + XRT group was 32 ± 8%, lower than the XRT group (54 ± 14%, *p* < 0.01). The early apoptotic rate of the UTMD + XRT group was 21.1 ± 3% at 48 h, higher than that of the XRT group (15.2 ± 4%). The tumor growth curve indicated that the tumor growth was inhibited in the UTMD + XRT group compared with other groups during 10 days of observation. In TUNEL experiment, the apoptotic cells of the UTMD + XRT group were higher than that of the XRT group (*p* < 0.05). The UTMD + XRT group had the lowest MVD value, but was not significantly different from other groups (*p* > 0.05). In addition, γH2AX increased due to the addition of UTMD to radiotherapy compared to XRT in immunohistochemistry (*p* < 0.05).

**Conclusions:**

Our study clearly demonstrated the enhanced destructive effect of UTMD combined with 4 Gy radiotherapy on glioblastoma. This could be partly achieved by the increased ability of DNA damage of tumor cells.

**Supplementary Information:**

The online version contains supplementary material available at 10.1007/s00432-021-03542-5.

## Introduction

Glioma is the most common and fatal type of primary brain tumors (Gusyatiner and Hegi 2018). Glioblastoma (GBM) accounts for 60–70% of these malignancies (Yang 2015) and has an average survival time of 12–15 months (Alexander and Cloughesy 2017). Due to the unique anatomical position and moderate radiosensitivity of tumors, radiotherapy (XRT) has been playing a leading role in the treatment of tumors. Nevertheless, it is well known that radiotherapy does not work well in glioma, especially in advanced-stage tumor GBM (Sulman 2017; Lieberman 2019), due to radiation resistance triggered by increasing dsDNA break (DSB) repair of cells (Carruthers (2018)) and angiogenesis (Garcia-Barros 2003). Therefore, many studies aim to optimize various methods to enhance sensitization of tumor radiotherapy.

To reduce radiation resistance and improve survival rate, many researchers have focused on developing innovative radiation sensitizers. Recent studies (Jing 2019; Shen 2016) have revealed that ultrasound-targeted microbubble destruction (UTMD) could cause damage to vessels in tumors. The ultrasound-guided local treatment is provided by microbubbles to increase the tumor kill and, therefore, allows potential dose de-escalation and normal tissue sparing (Czarnota 2015; Kaffas and Czarnota 2015; Cui 2017). The recent result suggests that the UTMD could effectively inhibit the growth of colon cancer in nude mice by blood vessel disruption and tumor tissue impairment (Huang 2013). According to the studies on the sensitivity of endothelial cells, the UTMD could considerably enhance the radiotherapy of various kinds of tumors, such as prostate, nasopharyngeal and breast cancers (Klein 2020; Deng 2018; Eisenbrey 2018). Apart from the broad spectrum of radiosensitization approach, we introduce a novel approach that can noticeably increase cell death when it is combined with radiotherapy. Physical forces generated by microbubbles combined with pulsed ultrasound were used to induce tumor death, instead of molecular biology techniques (Lin 2018).

This study aims to investigate the enhanced radiosensitization of UTMD in human malignant glioblastoma and its possible mechanism. More specifically, we hypothesized that it was effective to use lower dose of XRT and microbubbles by considering the potential evidence of biochemical and physiological mechanisms. To test this hypothesis, we managed to determine the best biophysical parameters of UTMD by examining the combined effects of UTMD and radiotherapy on tumor cell death. Because the tumoricidal effect of radiotherapy is copiously attributed to the induction of DSBs, we examined the expression of DSB-related proteins and microvascular densities (MVD) after the UTMD treatment or its combination with radiotherapy.

## Materials and methods

### Cell culture and animal tumor model

Cell lines were obtained from the American Type Culture Collection (ATCC, Manassas VA, USA). Human malignant glioblastoma U87MG cell lines were cultured in Eagle’s minimum essential medium (ATCC) supplemented with 10% fetal bovine serum (FBS, Gibco) and 1% penicillin/streptomycin (Sigma-Aldrich), and were exposed to 5% CO^2^ hepa-filtered air at 37 °C. 1.0 × 10^7^ U87MG cells were then injected subcutaneously to the right hind leg of the male BALB/c nude mice (aged 4–6 weeks, SLAC, Shanghai, China). Tumor size was continuously blindly determined by periodic caliper every 2–4 days and calculated using a modified ellipsoidal formula (volume = length × width^2^/2). It took nearly 4 weeks for the tumors to be ready for experiments by reaching a diameter of 8 mm (volume 100–300 mm^3^). Four experimental groups were compared: (A) no treatment (Control), (B) UTMD, (C) XRT, (D) UTMD + XRT (XRT was conducted immediately after UTMD). 24 nude mice were assigned to 1 of 4 groups in a stratified random-sampling manner according to the tumor diameter size. Animals were anesthetized during imaging and treatment. All research procedures were operated to minimize the nude mice’ suffering.

### Microbubble preparation

SonoVue™ (Bracco, Milan, Italy) microbubbles encapsulating sulfur hexafluoride gas (SF6) were made by following the manufacturer's guideline, with an average yield of 2.5 μm in diameter and a concentration between 1 and 5 × 10^8^ microbubbles/mL (SF6 8 µl/ml) (Lammertink 2016). In vitro, prior to ultrasound treatment, 500 µl of freshly made microbubbles were mixed with medium (0.01%, v/v) and was inverted for several minutes to make the microbubbles rise into the cells, thereby ensuring a close touch between the cell and the microbubbles. In vivo, microbubbles were resuscitated by jiggling the vial before injection each time and administered as a bolus injection of 0.1 mL (fresh made) through tail vein.

### Ultrasound treatment and radiation

An ultrasound treatment system (IntelectTranSport^®^ Ultrasound, Chattanooga, USA) with a planar 1 MHz probe was used to insonify tumor cells or tissues in to stimulate microbubbles in the sonoporation experiments. In vitro, after microbubbles were added to the six-well plate, cells for each group were treated with the 1 MHz probe for 1 min under the condition of duty cycle of 20% and peak negative pressure of 0.3 w/cm^2^. And then, cells were immediately irradiated using an irradiation cabinet (Siemens, Primus H, USA) after the ultrasound treatment. For in vitro experiments, X-rays were delivered at doses of 0, 2, 4, 6, 8, or 10 Gy at a dose rate of 300 cGy/min and an energy of 6 MV. In vivo, microbubbles were irrigated with normal saline through tail-vein catheter, and the tumors were promptly exposed to ultrasound for 1 min for the total treatment time of each sample. Peak negative pressure of 0.3 w/cm^2^ was applied by using a calibrated ultrasound transducer resulting in an average duty cycle of 20%, the same condition with the in vitro experiment. Tumors were immediately irradiated with ionizing irradiation right after the UTMD treatment.

### Clonogenic assay

The cells were located in 60 mm petri dish (1000/dish) and irradiated with different doses, with or without UTMD. Plates were incubated without interference for 2 weeks. Stained colonies were counted and recorded while consisting of > 50 cells. For the combinational therapy of UTMD and radiotherapy, the surviving fraction was normalized to the control group. A multi-target click mathematical model was used to simulate the surviving fraction (SF) curve of cells, with its associated equations and radiological parameters (Huaying 2016). Cell survival was plotted using the irradiation dose as the abscissa axis and the SF as the vertical axis. The average lethal dosage of cells (*D*_0_) and the quasi-field dosage (*D*_q_), which specified the repair ability of cells to sublethal injury, and extrapolation number (*N*) values were calculated according to the curve. SF was calculated using SF = 1 − (1 − exp − [*D*/*D*_0_])*N*; *D*_q_ = In*N*/(1/*D*_0_), and SER was calculated as SER = control group *D*_0_/treatment group *D*_0_. The software CalcuSyn2 (USA) was used to evaluate whether to sensitize radiotherapy. A general equation for dose–effect relationship was derived by Chou (1994) through mathematical induction using hundreds of enzyme kinetic models. It correlates the “Dose” and the “Effect” in the simplest possible form. We used the recommended symbols for describing synergism or antagonism in drug combination studies and analyzed with the combination index (CI) method to determine whether to sensitize radiotherapy**.**

### Apoptosis assay

Log-phase growing cells were treated with radiation with or without UTMD for 48 h. The cells were then mixed with staining buffer that contained Annexin V-FITC and PI on the basis of manufacturer’s instruction (BD Pharmingen), and flow cytometry was used to quantify apoptotic cells (FITC + /PI−).

### Western blot analysis

Proteins were extracted using RIPA buffer (Beyotime, China) from the tumor tissues. 50 μg tissue protein with loading buffer was loaded onto sodium dodecyl sulfate–polyacrylamide gel electrophoresis (SDS-PAGE). All primary antibodies (Cell Signaling Technology, MA, USA) including ATM, ATR, CHK1, CHK2, H2AX, p53, Phospho-BRCA1, Phospho-ATR, Phospho-CHK1, Phospho-CHK2, γH2AX, and Phospho-P53 were used at 1:500 to 1:1000 dilutions in this experiment, and beta-actin was used as the internal reference. Then, ImageJ software was used to analyze the bands.

### Immunohistochemistry

From each sample, sections were stained with hematoxylin and eosin (HE), γH2AX (Cell Signaling Technology, MA, USA), and cluster of differentiation 34 (CD34). The MVDs quantified by endothelial-specific CD34 staining was realized by counting the number of stained blood vessels in 4–5 fields of view, as described previously (Chabowski 2018). All immunohistological quantifications were performed by the same person for consistency.

### Terminal deoxynucleotidyl transferase-mediated dUTP nick and labeling (TUNEL) assay

According to the manufacturer’s instructions, we used In Situ Cell Death Detection Kit-Fluorescein (beyotime, China) to quantify TUNEL-positive cells of the xenograft tumor tissues through light and fluorescence microscopy. Percentage apoptosis was the ratio of the number of TUNEL-positive cells to the total number of nuclei (per 1000 tumor cells coming from 4 fields of view).

### Statistical analysis

Numerical results were reported as mean ± SD and analyzed in SPSS 17.0 soft package. All data analysis was demonstrated using Graph Pad Prism. Student’s *t* test and one-way analysis of variance were used to analyze inter-group and intra-group results, respectively.

## Results

### UTMD enhanced radiosensitivity in GBM cells

**Fig. 1:**
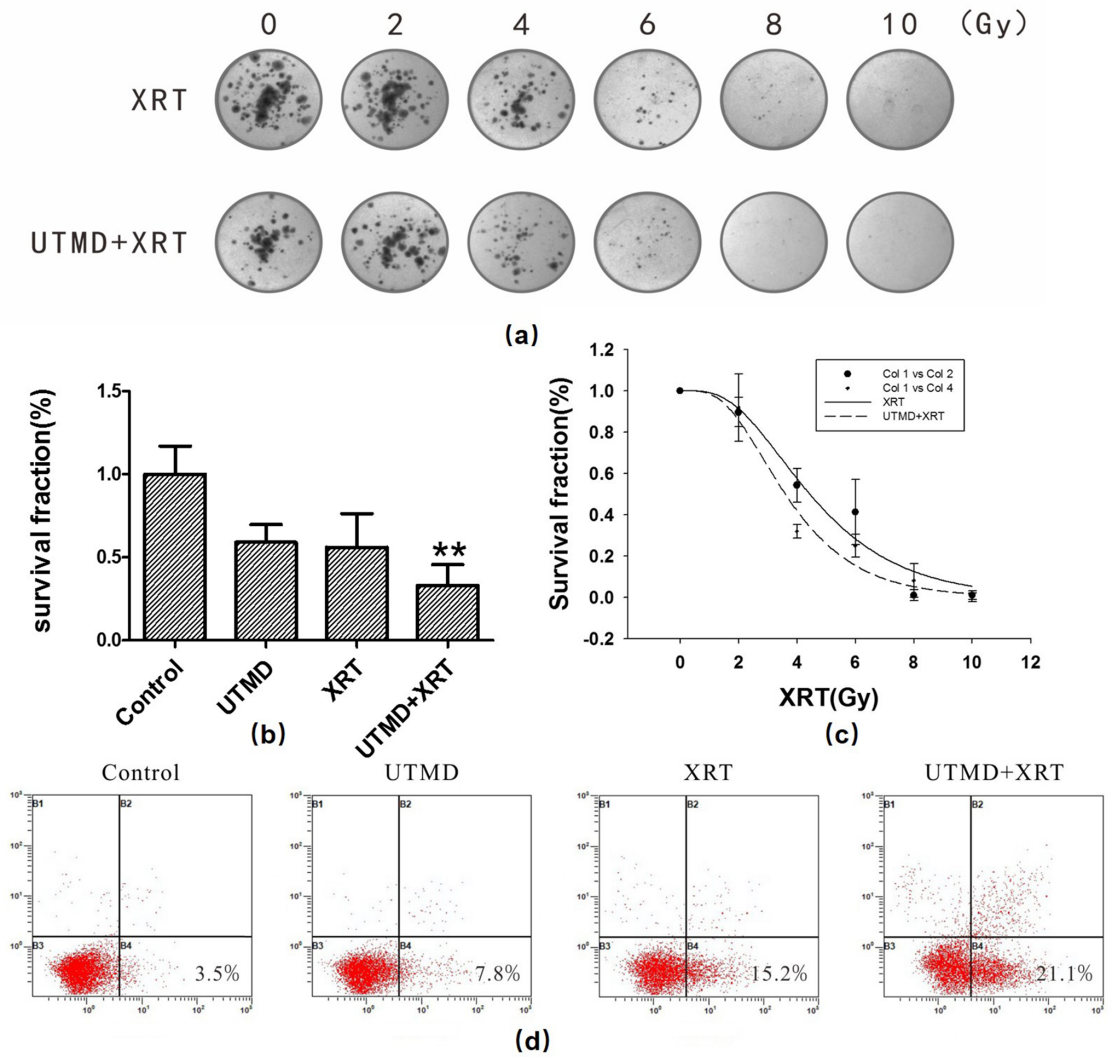
Clonogenic survival and cell apoptosis in U87MG cells. **a** Clonogenic survival of different irradiation doses for the XRT and UTMD + XRT groups. Representative images showed the surviving colonies. **b** Graphs showed the changes of clonogenic survival fraction. **p < 0.01 versus XRT group. **c** Cell survival curve and click multi-target fitting curve. Data present average results from three independent experiments (*n* = 3). **d** Representative images showing apoptosis in U87MG cells at 48 h after treatment. Percentage of the bottom right quadrant showed the average value of early apoptosis

To determine the potential radiosensitization effect of UTMD, the U87MG cells were irradiated with a dose of 0, 2, 4, 6, 8 or 10 Gy of XRT, and the cell survival was assessed using clonogenic assays. When XRT was higher than or equal to 4 Gy*,* the clonogenic rates of the U87MG cells in the UTMD + XRT group substantially decreased, and even became negligible (Fig. 1a). When the radiotherapy dose was 4 Gy, the clonogenic rate of cells in the UTMD + XRT group was statistically different from that of the XRT group (*p* < 0.01, Fig. 1b). The result revealed that UTMD combined with XRT could significantly increase the radiosensitivity of the U87MG cells (Fig. 1c). SF2, D0, and Dq decreased in the UTMD + XRT group, compared to the XRT group. The results calculated by CalcuSyn2 showed that when the dose was 4 Gy, CI was equal to 0.716, implying moderate synergism. Flow cytometry was used to detect the effect of UTMD on U87MG cell apoptosis. We found that U87MG cell apoptosis significantly increased after 48 h of treatment with XRT or UTMD + XRT. The average early apoptosis rate was 21.1 ± 3% at 48 h in the UTMD + XRT group and 7.8 ± 1% in the UTMD group (Fig. 1d). The difference between the two groups was statistically significant (*p* = 0.01). In the XRT group, the early apoptosis rate is 15.2 ± 4%, which is lower than the combined group but with no statistical difference.

### UTMD enhanced radiosensitivity in a GBM mouse model

**Fig. 2:**
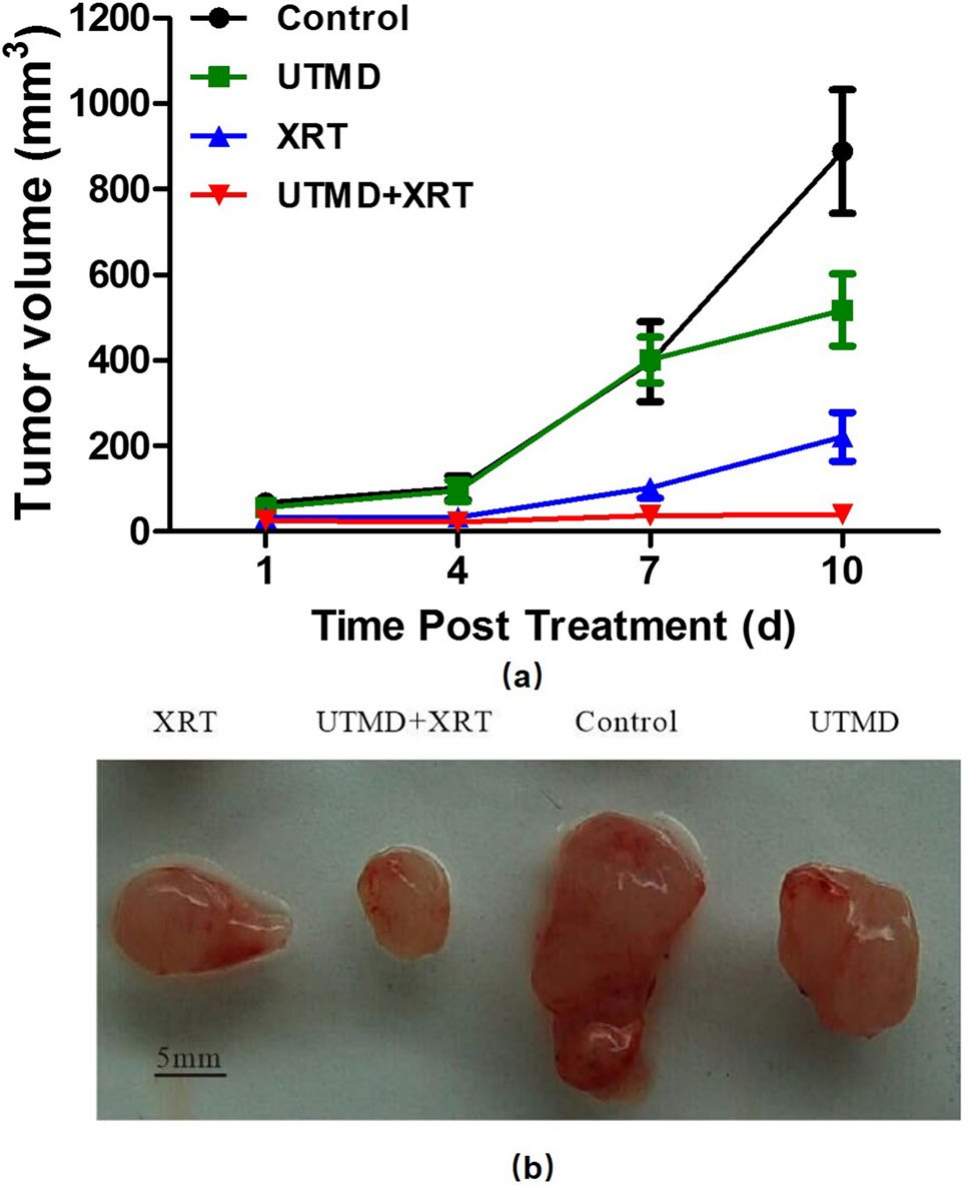
Effects of UTMD and radiation on the growth of U87MG xenografts in nude mice. **a** Tumor growth curves of different groups after treatment. **b** Photographs of excised xenografts from inoculated mice in different groups. Data are mean ± SD of the six mice per group (*n* = 6)

Figure 2 demonstrates the tumor volume growth difference among different treatment groups at day 1, 4, 7, and 10 after treatment. We found no significant difference in weight among the groups. Comparing to the control group, the overall tumor growth was greatly decreased when UTMD was administered jointly with radiotherapy (Fig. 2). By day 10, the tumor volume treated with UTMD + XRT was reduced to 8% of tumors treated by UTMD only and 18% of tumors treated by radiation only. However, the differences are not statistically significant among the four groups (*p* = 0.61) due to the large differences in each group.

**Fig. 3:**
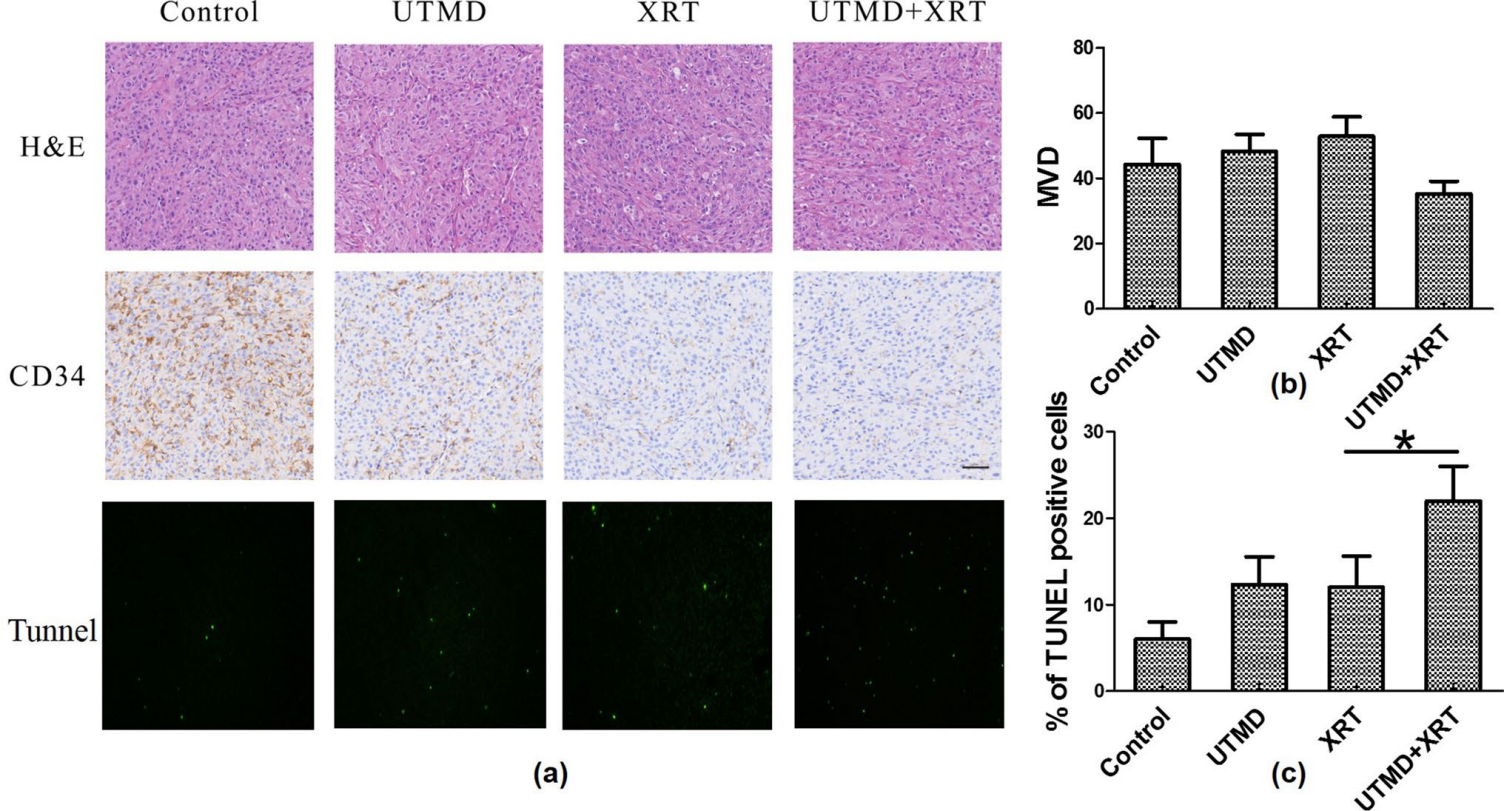
Histopathological findings of XRT combined with UTMD on tumor tissues. **a** Top row: the H&E staining of excited xenograft tissues showed typical glioblastoma with all otypic nuclear division and tumor giant cells; second row: representative images of CD34 stained tumour cross-sections, The scale bars represent 50 μm; Third row: representative images of apoptosis cells in tunnel experiment (× 100). **b** MVD (microvasculature density) comparison in different groups (*n* = 6). **c** Comparison of cell apoptosis in tissues of four groups of rats (*n* = 6), *p < 0.05 versus XRT groups

Figure 3a exhibits representative images of tunnel, HE- and CD34-stained tumor cross-sections. Qualitative reactions were observed in the treated and non-treated samples. MVD detection was based on quantitative CD34 staining. As shown in Fig. 3b, both radiation and UTMD increased CD34 expression, while the joint treatment reduced it. Analysis of CD34 expression indicated that the numbers of vessel significantly decreased under the joint treatment. However, no significant difference was observed in the expressions of CD34 among different groups (*p* > 0.05).

As presented in Fig. 3c, the percent of apoptosis cells per field of tumor tissue in the UTMD + XRT group was 22 ± 4%, while the XRT group was 12 ± 3.6%. The apoptosis rate in the UTMD + XRT group was significantly higher than that of the XRT group, and the difference was statistically significant (*p* < 0.05).

### Effect of radiation and UTMD on the expression of DNA damage and repair-related proteins

**Fig. 4:**
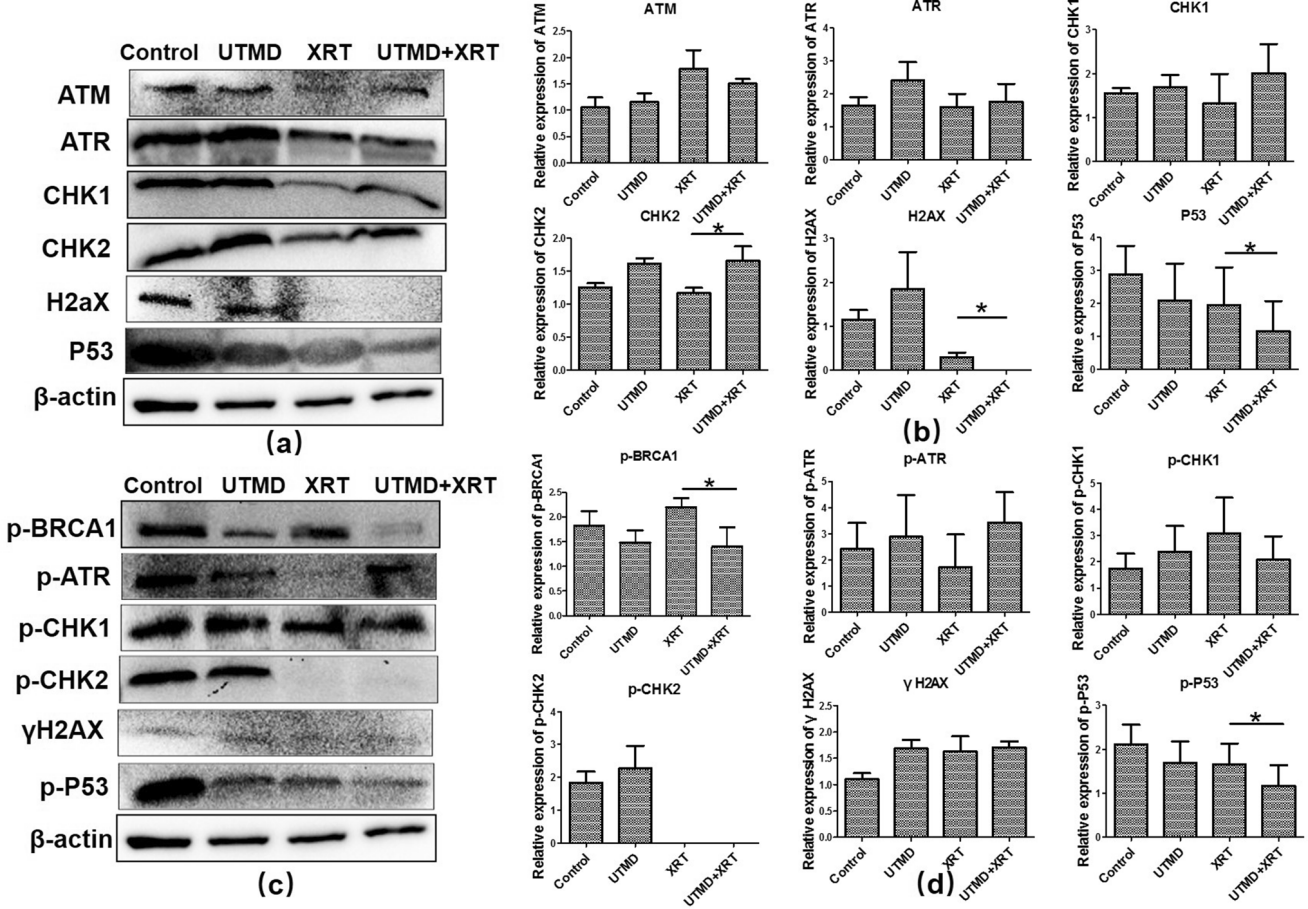
Western blot analysis showing the effects of UTMD combined with XRT on DSB repair-related proteins on xenograft tumor tissues. **a** Representative western blots showing the effects of UTMD on expression of ATM, ATR, CHK1, CHK2, H2AX, P53. **b** Graphs showing changes of protein expression in different groups. **c** Representative western blots showing the effects of UTMD on expression of Phospho-ATR, Phospho-BRCA1, Phospho-CHK1, Phospho-CHK2, γH2AX and Phospho-P53 (**d**) Graphs showing changes of protein expression among different groups. β-action was included as a loading control. Data represent average results from three independent experiments; SD signifies standard deviation (*n* = 3), *p < 0.05 versus XRT groups

**Fig. 5:**
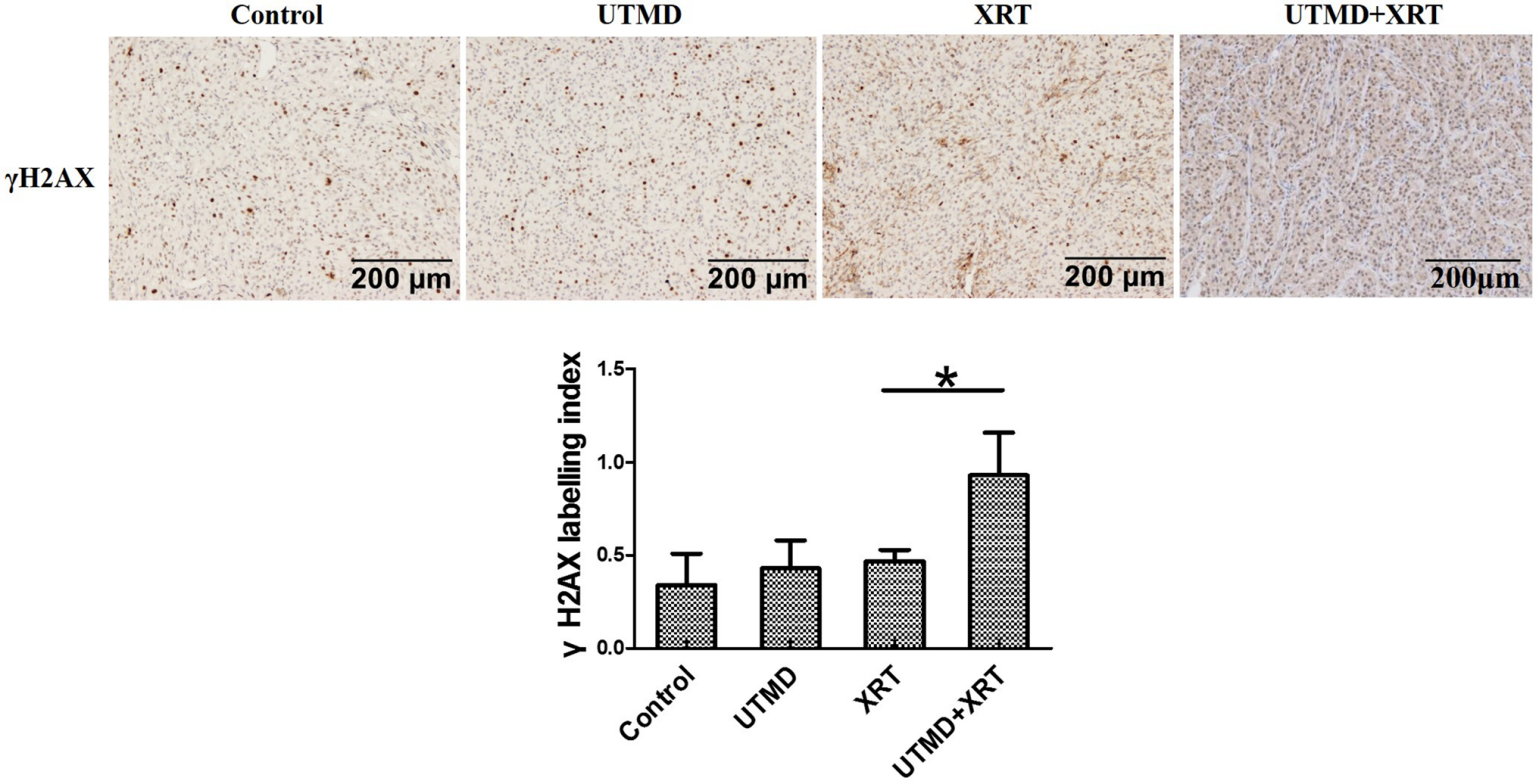
γH2AX labeling of tumor sections. **a** Representative images of γH2AX stained tumour cross-sections, The scale bars represent 200 μm. **b** γH2AX labeling comparison in different groups (*n* = 6), *p < 0.05 versus XRT groups

Figure 4a, b shows that the expression of CHK2 of tumor tissue increased, while H2AX and P53 decreased in the UTMD + XRT group, which was statistically different from the XRT group. Figure 4c, d demonstrates that the phosphorylation expression of BRCA1, CHK1, and P53 significantly decreased when the U87MG cells were treated by UTMD combined with radiation, compared with treated by XRT alone. Meanwhile, we added γH2AX immunohistochemical index of tissue samples. A significant increase in γH2AX labeling index was also examined when comparing single 4 Gy treatment with the joint treatment of UTMD with XRT (Fig. 5).

## Discussion

In this study, we investigated whether UTMD can enhance radiotherapy on the GBM model*.* We found that both levels of tumor cell death and damage to blood vessels increased when tumors were exposed to UTMD combined with radiation, which was consistent with previous studies in bladder and prostate tumor (Tran 2012; Kim 2013). As to radiotherapy, cytotoxicity or targeting drugs in a variable period of treatments tends to induce drug resistance on account of genetic heterogeneity and tumor instability. Regarding local responses, UTMD has been proved to be an adjuvant therapy of antitumor that can improve the utility of chemotherapy and boron neutron capture therapy (BNCT) in GBM (Dong 2018; Fan 2019). Previous research found that sensitization of GBM cells to radiation was regulated through glucose metabolism (Shen 2015). In a study on Asmase + / + astrocytes, Nofiele found that cell survival decreased from 56% after 2 Gy XRT alone and from 17% after UTMD alone, to 5% when they are combined (Nofiele 2013). In our study, using animal models in conjunction with U87MG, we observed that adjuvant UTMD had an additive effect on radiotherapy and established a significant new perspective on UTMD in GBM.

We also observed that radiosensitization was effective even with the clinically recommended microbubble concentration of 10 mL/kg, which is consistent with previous research (Briggs 2014). Based on our experimental results, the optimal microbubble concentration is 5 ml/kg with ultrasound pressure at 1 MHz and radiation dose at 4 Gy, while most previous studies suggested not only at 8 Gy (Lai 2016; Kaffas 2018a), but also at 2–6 Gy (Lammertink 2016; Kaffas 2014; Daecher 2017). Whereas the results from the experiments demonstrated the effects of the UTMD in combination with single doses of radiation, uncertainty remains with regard to the mechanism underlying sensitive tumor responses. Recent studies have suggested that large single doses of radiation (8–10 Gy) cause endothelial cell death through a ceramide-dependent mechanism (Kolesnick and Fuks 2003). The effects of ceramide dependence may lead to the lethal damage that accounts for tumor destruction. However, joint effects of low radiation doses and UTMD are likely relevant to other microbubbles or radiation-based tumor damage. More specifically, while high doses (> 8 Gy) release sufficient quantities of ceramide to cause endothelial cell death, radiation doses lower than 6 Gy do not activate adequate ceramide to prompt ceramide-induced cell death (Czarnota 2012). When ceramide is released following UTMD, it is not sufficient to quickly activate and wide spread cell death for vascular shut-down when XRT is used alone. Effects of 4 Gy treatment combined with UTMD were obvious compared with either treatment alone, as shown in the previous studies (Daecher 2017; Kaffas et al. 2013). As regard to clinical study, ultrasound pressure and microbubble concentration would need to be adjusted according to the lowest fixed radiation required.

The primary mechanism of ionizing radiation is to trigger cancer cell death directly by DNA disruption. The second one is involved in endothelial cell injury and death induced by radiation within the tumor microvasculature, which indirectly leads to cancer cell death (Kaffas 2018b). Previous studies have indicated that when the ionized radiation was used alone, the up-regulation of proteins associated with the DNA damage would be induced, leading to less apoptosis (Santivasi and Xia 2014; Xiao 2019). The γH2AX, a histone subtype related with DNA damage (Al-Mahrouki 2014), was up-regulated, while phospho-BRCA1, phospho-CHK1, and phospho-P53 were down-regulated by UTMD combined with radiotherapy. It indicates that the radiotherapy sensitization effect of UTMD on GBM was at least partially achieved by inhibiting the DSB repair ability of tumor cells. The expression of CD34, an endothelial cell marker, was the same as the existence of endothelial cells, and was utilized to define the structure and properties of the vasculature (Deng 2018). MVD assessments of CD34 cannot distinguish the perfused vessels from the non-perfused ones (Chabowski 2018). Using electron microscopy, the absence of nucleus membrane, chromatin condensation, and mitochondrial vacuolation were also observed in the UTMD + XRT group as pathological changes, whereas other groups rarely happen. These findings imply that the oscillation, collapse, and inertial of bubbles in microvessels or immediate cavitation in the process of US sonication can disrupt the tumor microvessels and cause tumor cell injury and lysis. Our study suggested that the tumor tissue from intravenous microbubbles combined with ultrasonic exposure treatment decreased expression of CD34 compared with other groups, and the treatment of intravenous microbubbles combined with ultrasonic exposure may inhibit angiogenesis. However, it showed no significant difference from other groups in our study. It is possibly because the animal scale was too small. Moreover, the check time point warrants further research since previous studies found that the MVD was different from other groups in mouse tumor model after 12 h and 24 h (Lai 2016; Kaffas 2018a).

Some limitations and future research directions are worth discussing. Our experiments were conducted with xenografts in nude mice. It demands adjustments to amplification for nude mice owing to vascularity can be heterogeneous. Therefore, further studies are needed to optimize the type of ultrasonic contrast agent and injection time in animal model in situ. In addition, we aim to expand this research in the future to discover the mechanisms of both ultrasonic acoustic cavitation and ceramide-dependent entendothelial cell death.

## Conclusions

This research revealed that the UTMD could be a safe and feasible technique to slow the GBM growth in vitro and in vivo. Although the application of UTMD in GBM tumor therapy is still in the experimental stage, we believe that this new technology would potentially help in conducting radiation treatments with lower doses of radiation, and sparing patients from normal tissue damage.

## Supplementary Information

Below is the link to the electronic supplementary material.

Effects of UTMD and XRT on cell cycling distribution of U87MG cells. (a) Representative results of the flow cytometry analysis with U87MG cells in different treatments after 48h. (b) Graphs showing the percentage for each cell cycle in U87MG cells of each group. Data present average results from three independent experiments (n=3) (JPG 218 KB)
