# Supplementary figures and images for: Using ultrasound-targeted microbubble destruction to enhance radiotherapy of glioblastoma
Source: J Cancer Res Clin Oncol. 2021 Feb 6;147(5):1355–63. doi: 10.1007/s00432-021-03542-5 (PMC8021517; doi:10.1007/s00432-021-03542-5)

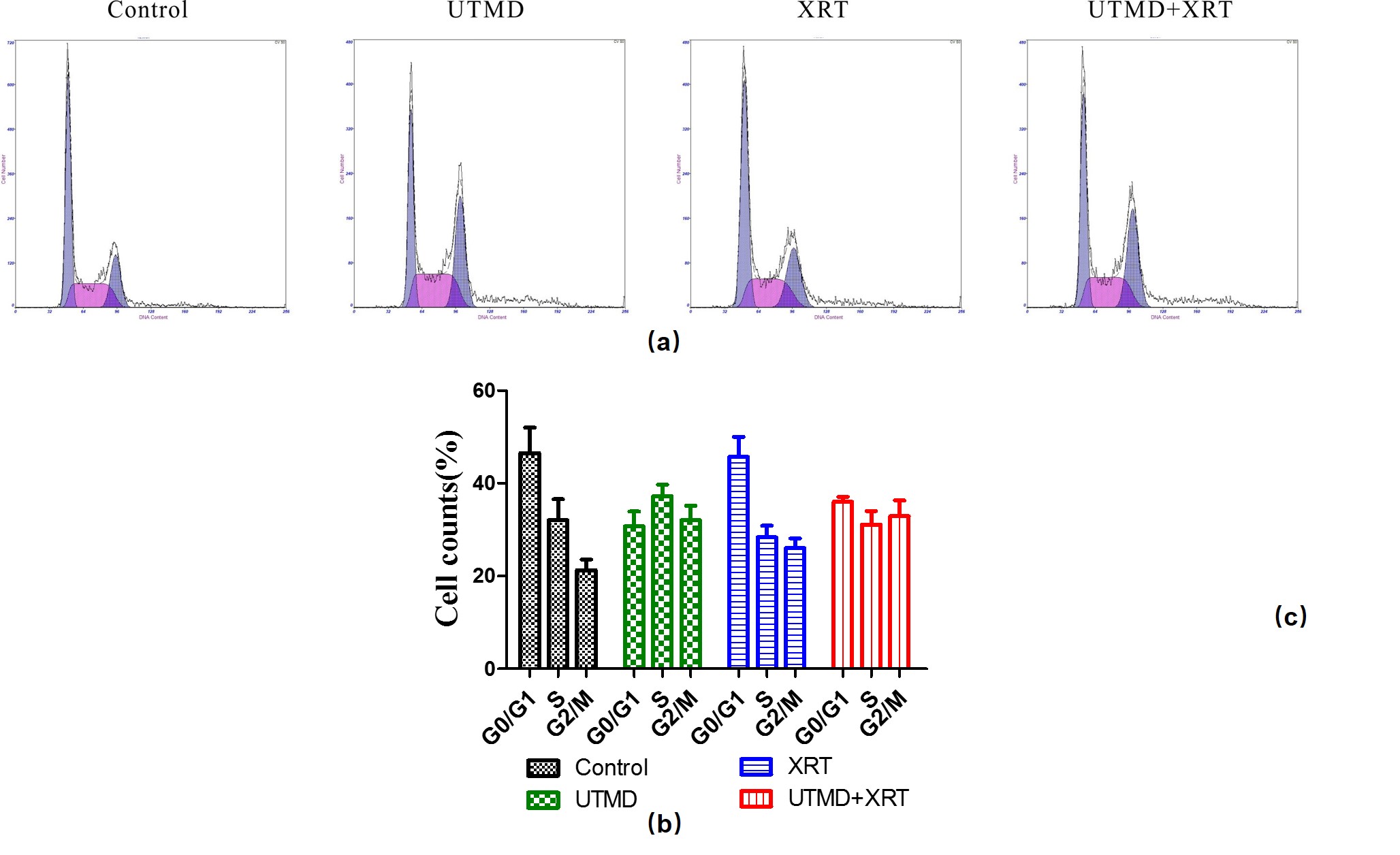

Supplement: Supplementary file 1 — Effects of UTMD and XRT on cell cycling distribution of U87MG cells. (a) Representative results of the flow cytometry analysis with U87MG cells in different treatments after 48h. (b) Graphs showing the percentage for each cell cycle in U87MG cells of each group. Data present average results from three independent experiments (n=3) (JPG 218 KB) [file 432_2021_3542_MOESM1_ESM.jpg]
